# Expression of T cell-related lncRNAs in multiple sclerosis

**DOI:** 10.3389/fgene.2022.967157

**Published:** 2022-08-26

**Authors:** Maryam Dadyar, Bashdar Mahmud Hussen, Solat Eslami, Mohammad Taheri, Farhad Emadi, Soudeh Ghafouri-Fard, Arezou Sayad

**Affiliations:** ^1^ Department of Medical Genetics, School of Medicine, Shahid Beheshti University of Medical Sciences, Tehran, Iran; ^2^ Department of Pharmacognosy, College of Pharmacy, Hawler Medical University, Kurdistan Region, Erbil, Iraq; ^3^ Center of Research and Strategic Studies, Lebanese French University, Kurdistan Region, Erbil, Iraq; ^4^ Department of Medical Biotechnology, School of Medicine, Alborz University of Medical Sciences, Karaj, Iran; ^5^ Dietary Supplements and Probiotic Research Center, Alborz University of Medical Sciences, Karaj, Iran; ^6^ Institute of Human Genetics, Jena University Hospital, Jena, Germany; ^7^ Skull Base Research Center, Shahid Beheshti University of Medical Sciences, Tehran, Iran

**Keywords:** FLICR, NEST, RMRP, TH2-LCR, lncRNA, multiple sclerosis, T cell

## Abstract

Long non-coding RNAs (lncRNAs) have been demonstrated to in the pathophysiology of multiple sclerosis (MS). In order to appraise the role of T cell-related lncRNAs in this disorder, we assessed expressions of NEST, RMRP, TH2-LCR, MAFTRR and FLICR in MS patients and healthy individuals. We detected significant difference in the expression of RMRP and FLICR between cases and controls. There were substantial correlations between expressions of NEST, RMRP, TH2-LCR, MAFTRR and FLICR lncRNAs among patients, but not controls. The strongest correlations were found between RMRP and TH2-LCR, and between MAFTRR and RMRP with correlation coefficients of 0.69 and 0.59, respectively. ROC curve analysis revealed appropriate power of FLICR in differentiating between MS patients and healthy controls (AUC value = 0.84). Expression of NEST lncRNA was positively correlated with disease duration in MS patients, but negatively correlated with age at onset. In brief, we reported dysregulation of two T cell-related lncRNAs in MS patients and proposed FLICR as a putative marker for this disorder.

## Introduction

Multiple sclerosis (MS) is an autoimmune condition in which the underlying cause is not completely understood. Autoimmune reactions may occur as a result of inappropriate function of regulatory T cells (Tregs). This population of T cells expresses Foxp3 and low level of CD127 (Seddiki et al., 2006). Several genes associated with MS such as CD25, CTLA4, CD127, IL-10 have been shown to be related to Treg function or differentiation (ANZgene, 2009; Rioux et al., 2009). Numerous studies have reported abnormal function or quantities of Tregs in blood (Viglietta et al., 2004; Venken et al., 2008; Dalla Libera et al., 2011; Rodi et al., 2016), central nervous system (CNS) lesions and cerebrospinal fluid (Fritzsching et al., 2011) of MS patients. Notably, expression levels of Foxp3 have been revealed to be diminished in Tregs of MS patients (Venken et al., 2008; Frisullo et al., 2009). Moreover, their ability in inhibition of T cell response to myelin basic protein has been diminished (Haas et al., 2005; Venken et al., 2007). A recent study has reported decline in the number of resting and increase in activated CD4^+^CD25+FOXP3+Tregs in MS patients (Verma et al., 2021). Cumulatively, abnormal function of Tregs is a predominant finding in MS patients.

Recent studies have indicated the importance of non-coding RNAs in the regulation of Treg function and their plasticity as well as commitment of Treg lineage (Ghafouri-Fard and Taheri, 2020; Luo and Wang, 2020; Jalaiei et al., 2021; Taheri et al., 2021). A number of newly identified long non-coding RNAs (lncRNAs), namely FLICR (FOXP3 Regulating Long Intergenic Non-Coding RNA), MAFTRR (MAF Transcriptional Regulator RNA), NEST (IFNG-AS1), RMRP (RNA Component Of Mitochondrial RNA Processing Endoribonuclease) and TH2-LCR (Th2 Cytokine Locus Control Region) are supposed to affect function of Tregs. However, experimental data for confirmation of their effect in the pathogenesis of MS is missing. We conducted this study to evaluate expression of these lncRNAs in the peripheral blood of MS patients in comparison with healthy persons.

## Materials and methods

### Study participants

The study included 12 male MS patients and 38 female MS patients. Additionally, 50 age and sex matched healthy individuals were recruited as controls. The latter group of individuals had no history of neuropsychiatric or immune-mediated diseases. MS patients were diagnosed based on the revised McDonald criteria (Polman et al., 2011). Relapsing-remitting MS was diagnosed based on the presence of at least two separate areas of damage in the CNS that have happened at dissimilar points in time. MRI results, history of symptoms and findings on the neurological examination were used for diagnosis. Informed consent was obtained from all MS patients and healthy controls. The study protocol was approved by the ethical committee of Shahid Beheshti University of Medical Sciences (IR.SBMU.MSP.REC.1400.562). General data of MS patients and controls is summarized in Table 1.

**TABLE 1:** General parameters of study participants.

Study groups	Parameters	Values	
Patients	Sex (number)	Male	12
		Female	38
	Age (Years, mean ± SD)	Male	41.08 ± 9.5
		Female	39.13 ± 8.99
	Disease duration (Years, mean ± SD)	Male	3.75 ± 2.3
		Female	10.34 ± 6.34
	Age of onset (Years, mean ± SD)	Male	37.33 ± 10.49
		Female	28.76 ± 8.84
	EDSS Score	Male	2.62 ± 0.77
		Female	2.3 ± 1.24
Controls	Sex (number)	Male	12
		Female	38

### Expression studies

Four milliliters of whole peripheral blood were gathered from cases and controls. Blood specimens were used for RNA extraction using the Hybrid-RTM blood RNA extraction kit (GeneAll Biotechnology Co Ltd., South Koera). Then, cDNA was produced using the extracted RNA and a commercial Reverse Transcription kit (Applied Biosystems). Expressions of NEST, RMRP, TH2-LCR, MAFTRR and FLICR were quantified using the primers listed in Table 2. B2M was used as the reference gene for qPCR.

**TABLE 2:** Primer sequences.

Gene	Sequence 5→3	Primer length (bp)
*B2M*	F- AGA​TGA​GTA​TGC​CTG​CCG​TG	20
	R- GCG​GCA​TCT​TCA​AAC​CTC​CA	20
*FLICR*	F- GGG CTT TTC CAG AAG GGT CT	20
	R- AGC CCA GGG TTC TAG TCG	18
*MAFTRR*	F- CTG AAG GGA CAG GAC GGA CAA C	22
	R- GGG GAA AAC CTG GAA AGA GGG A	22
*NEST*	F- AGC TGA TGA TGG TGG CAA TCT	21
	R- TGA CTT CTC CTC CAG CGT TTT	21
*RMRP*	F- GTA GAC ATT CCC CGC TTC CCA	21
	R- GAG AAT GAG CCC CGT GTG GTT	21
*TH2-LCR*	F- GCT CCC CAG GCT TTT GAG ATA	21
	R- TGG TGA TGC TGA AGG GAG AC	20

### Statistical analysis

GraphPad Prism version 9.0 (La Jolla, CA, United States) was used for data assessment. Expression levels of five lncRNAs, namely NEST, RMRP, TH2-LCR, MAFTRR, and FLICR were compared between MS patients and healthy controls using the comparative–delta Ct method. The normal/gaussian distribution of the values was assessed using the Shapiro-Wilk test. Mann-Whitney U test was applied to recognize differentially expressed genes between MS patients and controls. Two-way ANOVA test was applied to examine the effects of disease and gender on expressions of lncRNAs.

Correlations between expression levels NEST, RMRP, TH2-LCR, MAFTRR and FLICR were measured using Spearman’s rank correlation coefficient since expression data was not normally distributed. Correlations between expressions of lncRNAs and demographic/clinical data such as age, disease duration, age at onset and EDSS were assessed using Spearman’s rank correlation coefficients.

Receiver operating characteristic (ROC) curve was illustrated to value the diagnostic power of expression levels of differentially expressed genes. *p* value < 0.05 was considered as significant.

## Results

### Expression data

In the current study, we assessed expression of five lncRNAs, namely NEST, RMRP, TH2-LCR, MAFTRR and FLICR. Table 3 shows the information about selected lncRNAs.

**TABLE 3:** Information about selected lncRNAs.

Name/Gene ID	Accession number	Location	Official full name
IFNG-AS1 (NEST)	NR_104124.1	12q15	IFNG antisense RNA 1
	NR_104125.1		
RMRP	NR_003051.3	9p13.3	RNA component of mitochondrial RNA processing endoribonuclease
TH2LCRR (TH2-LCR)	NR_132124.1	5q31.1	T helper type 2 locus control region associated RNA
	NR_132125.1		
	NR_132126.1		
MAFTRR	NR_104663.1	16q23.2	MAF transcriptional regulator RNA
FLICR	NR_147988.1	Xp11.23	FOXP3 regulating long intergenic non-coding RNA

**FIGURE 1:**
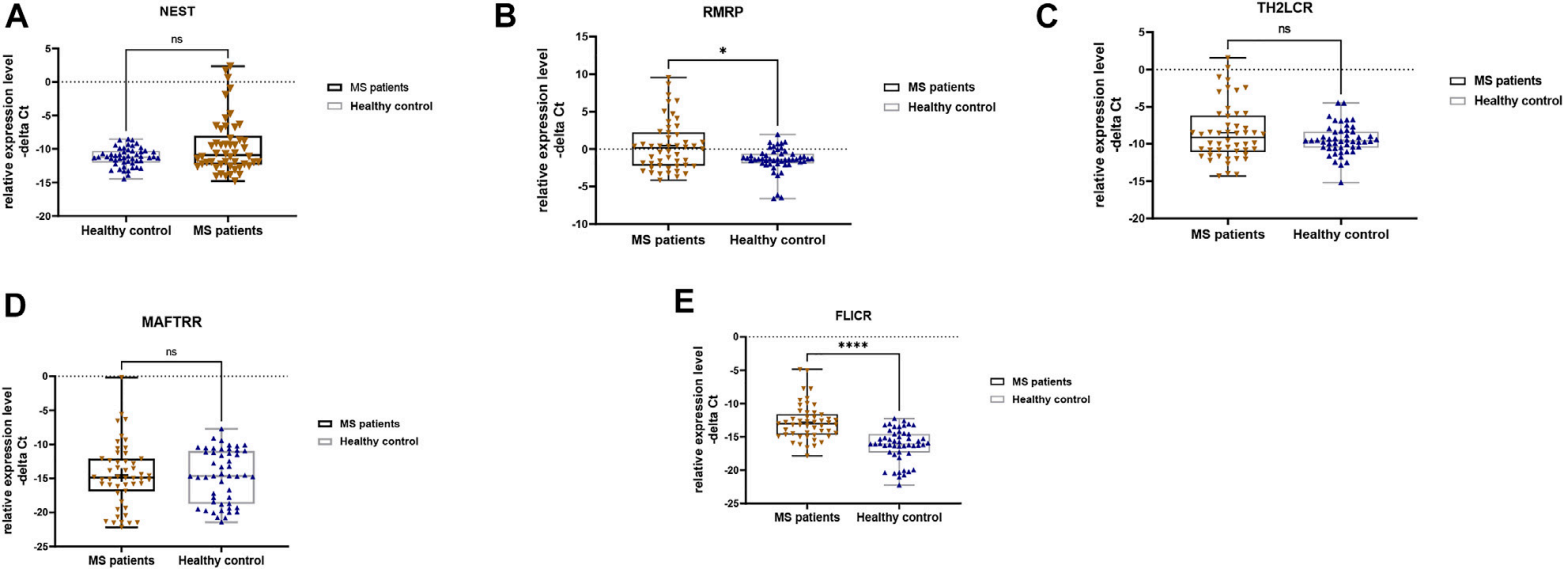
Relative expression levels of five lncRNAs in total multiple sclerosis (MS) patients and controls as measured by–delta Ct values **(A–E)**. Mann-Whitney U test was applied to find differentially expressed genes between cases and controls (∗∗*p* value <0.01 and ∗∗∗∗*p* value <0.0001).

Expression levels of RMRP and FLICR were different between MS patients and controls (Figure 1).

We detected significant effect of group (disease) factor on expression levels of RMRP and FLICR. However, gender factor had no significant effect on expressions of these lncRNAs, which means that there is no significant difference in the expression of these lncRNAs between males and females. Moreover, the interaction of gender and group had no effect on expressions of any of the studied genes (Table 4). Therefore, we did not perform post hoc tests for multiple comparisons.

**TABLE 4:** Graphpad prism output from analysis of effect of Group and Gender (Tests of Between-Subjects Effects) on expression of studied genes in cases compared to healthy controls.

Source of variation	Group effect			Gender effect			Interactions		
	SS^a^	F^b^	*p* Value	SS	F	*p* Value	SS	F	*p* Value
NEST	31.28	3.03	0.072	22.9	2.42	0.12	7.18	0.75	0.38
RMRP	58.05	8.21	0.005*	3.74	0.53	0.46	0.48	0.068	0.79
TH2-LCR	34.13	3.67	0.058	0.22	0.023	0.87	9.85	1.059	0.3
MAFTRR	0.13	0.007	0.93	3.33	0.17	0.67	0.61	0.03	0.85
FLICR	233.1	34.11	<0.0001*	6.92	1.01	0.31	0.50	0.07	0.78

a Sum of Squares.

b F of Variance.

**FIGURE 2:**
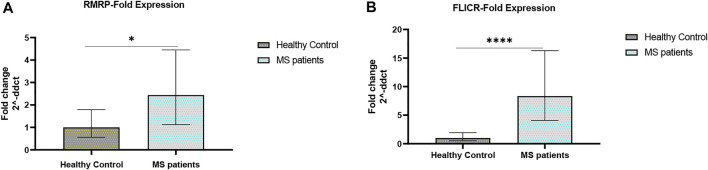
Fold changes of RMRP and FLICR expression **(A,B)** in MS patients versus healthy controls (∗*p* value <0.05, and ∗∗∗∗*p* value <0.0001).

Expression levels of RMRP and FLICR were significantly elevated in MS patients compared with controls (Figure 2).

Our data revealed significant correlations between expression levels of NEST, RMRP, TH2-LCR, MAFTRR and FLICR lncRNAs among patients, but not controls. The strongest correlations were found between RMRP and TH2-LCR, and between MAFTRR and RMRP with correlation coefficients of 0.69 and 0.59, respectively (Tables 5, 6).

**TABLE 5:** Spearman’s correlation between expression levels of lncRNAs among MS patients and healthy subjects (* *p*-Value at a significance level of *p* < 0.05.** *p*-Value at a significance level of *p* < 0.001).

	RMRP		TH2-LCR		MAFTRR		FLICR	
	Patients	Controls	Patients	Controls	Patients	Controls	Patients	Controls
NEST	0.4*	0.25	0.37*	0.21	0.38*	0.04	0.49**	0.046
RMRP			0.69**	0.016	0.59**	0.15	0.49**	-0.24
TH2-LCR					0.51**	0.08	0.44*	0.23
MAFTRR							0.42*	-0.13

**TABLE 6:** The results of ROC curve analyses for two differentially expressed lncRNAs in patients with MS disease.

FLICR				RMRP			
AUC±SD	Sensitivity	Specificity	*p* value	AUC±SD	Sensitivity	Specificity	*p* value
0.84 ± 0.03	0.86	0.72	<0.0001	0.63 ± 0.05	0.5	0.88	0.02

**FIGURE 3:**
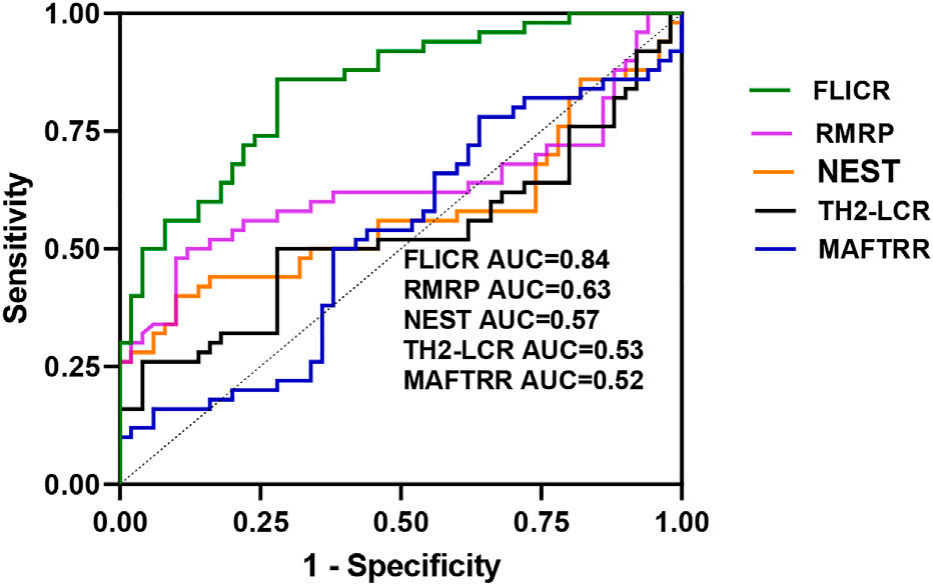
ROC curves of five lncRNAs levels in patients with multiple sclerosis.

ROC curve analysis revealed appropriate power of FLICR in differentiating between MS patients and healthy controls (AUC value=0.84). RMRP, NEST, TH2-LCR and MAFTRR had AUC values of 0.63, 0.57, 0.53 and 0.52, respectively (Figure 3).

Sensitivity and specificity values for two differentially expressed genes between cases and controls, i.e., FLICR and RMRP are shown in Tables 5, 6.

Expression of NEST lncRNA was positively correlated with disease duration in MS patients, but negatively correlated with age at onset (Table 7).

**TABLE 7:** The results of Spearman’s rank correlation between expressions of five lncRNAs and clinical data.

	Age	Disease duration	Age at onset	EDSS
NEST	−0.207	0.289*	−0.32*	0.09
RMRP	−0.076	−0.03	−0.04	−0.03
TH2-LCR	−0.183	−0.121	−0.1	0.047
MAFTRR	−0.058	0.014	−0.08	−0.04
FLICR	−0.157	0.194	−0.26	0.094

*Significance level of *p* < 0.05.

**Significance level of *p* < 0.001.

Disease duration was classified into 3 ranges (1–5, 6–10 and more than 4 years).

EDSS, scores was classified into 2 ranges (1-2 and 3–5).

## Discussion

Tregs have important functions in the pathoetiology of MS. Alterations in the frequency and suppressive effects of Tregs in MS patients is implicated with the evolution and exacerbation of MS (Kimura, 2020). Therefore, genes that regulate function of Tregs are potentially involved in the pathoetiology of this disorder. We compared expression of five Treg-associated lncRNAs between MS patients and healthy persons. We noticed higher expression levels of RMRP and FLICR in MS patients compared with controls. Consistent with our findings, a recent investigation has reported significant up-regulation of RMRP in drug-naïve relapsing remitting MS patients compared to healthy persons (Rahmani et al., 2021). However, expression of this lncRNA has been lower in IFNβ-1α-treated patients compared with drug-naïve patients (Rahmani et al., 2021). FLICR has a role as negative regulator of FOXP3 *in cis*. Notably, it is expressed in Tregs (Zemmour et al., 2017). Its role in the regulation of FOXP3 expression is exerted through cooperation with TGFβ and IL-2 signaling (Zemmour et al., 2017). Up-regulation of this lncRNA in MS patients might reflect abnormal activity of at least a subgroup of Tregs in these patients.

Besides, our data supported significant correlations between expression levels of NEST, RMRP, TH2-LCR, MAFTRR and FLICR lncRNAs among MS patients, but not controls. This finding indicates the presence of a disease-related regulatory or interactive mechanism among these lncRNAs. Therefore, interruption of this network might influence pathogenesis of MS.

Consistent with substantial up-regulation of FLICR in MS patients, ROC curve analysis revealed appropriate power of FLICR in differentiating between MS patients and healthy controls, suggesting this lncRNA as a possible peripheral marker of MS.

Finally, we detected positive correlation between expression of NEST lncRNA and disease duration in MS patients, but its expression was inversely correlated with age at onset of MS. NEST has been previously reported to participate in the epigenetic activation of the *IFNG* locus (Gomez et al., 2013). IFN-γ is a key cytokine detected in MS lesions. Moreover, IFN-γ levels are impressively up-regulated during MS activity. Besides, IFN-γ–producing Th1 responses are associated with inflammation both in MS patients and animal models of this disorder (Zamvil and Steinman, 1990; Liblau et al., 1995).

Taken together, we reported up-regulation of two Treg-related lncRNAs in MS patients and suggested one of these lncRNAs as a putative marker for MS. We recommend conduction of additional studies for verification of these results.

## Data Availability

The original contributions presented in the study are included in the article/supplementary materials, further inquiries can be directed to the corresponding authors.
